# Supervised Machine-Learning Enables Segmentation and Evaluation of Heterogeneous Post-treatment Changes in Multi-Parametric MRI of Soft-Tissue Sarcoma

**DOI:** 10.3389/fonc.2019.00941

**Published:** 2019-10-10

**Authors:** Matthew D. Blackledge, Jessica M. Winfield, Aisha Miah, Dirk Strauss, Khin Thway, Veronica A. Morgan, David J. Collins, Dow-Mu Koh, Martin O. Leach, Christina Messiou

**Affiliations:** ^1^Cancer Research UK Cancer Imaging Centre, Division of Radiotherapy and Imaging, The Institute of Cancer Research, London, United Kingdom; ^2^Department of Radiology, The Royal Marsden NHS Foundation Trust, Sutton, United Kingdom; ^3^Sarcoma Unit, Department of Radiotherapy and Physics, The Royal Marsden NHS Foundation Trust, London, United Kingdom; ^4^Division of Radiotherapy and Imaging, The Institute of Cancer Research, London, United Kingdom; ^5^Department of Surgery, The Royal Marsden NHS Foundation Trust, London, United Kingdom; ^6^Department of Histopathology, The Royal Marsden NHS Foundation Trust, London, United Kingdom

**Keywords:** magnetic resonance imaging, soft-tissue sarcoma, artificial intelligence, cancer heterogeneity, radiotherapy, imaging biomarkers

## Abstract

**Background:** Multi-parametric MRI provides non-invasive methods for response assessment of soft-tissue sarcoma (STS) from non-surgical treatments. However, evaluation of MRI parameters over the whole tumor volume may not reveal the full extent of post-treatment changes as STS tumors are often highly heterogeneous, including cellular tumor, fat, necrosis, and cystic tissue compartments. In this pilot study, we investigate the use of machine-learning approaches to automatically delineate tissue compartments in STS, and use this approach to monitor post-radiotherapy changes.

**Methods:** Eighteen patients with retroperitoneal sarcoma were imaged using multi-parametric MRI; 8/18 received a follow-up imaging study 2–4 weeks after pre-operative radiotherapy. Eight commonly-used supervised machine-learning techniques were optimized for classifying pixels into one of five tissue sub-types using an exhaustive cross-validation approach and expert-defined regions of interest as a gold standard. Final pixel classification was smoothed using a Markov Random Field (MRF) prior distribution on the final machine-learning models.

**Findings:** 5/8 machine-learning techniques demonstrated high median cross-validation accuracies (82.2%, range 80.5–82.5%) with no significant difference between these five methods. One technique was selected (Naïve-Bayes) due to its relatively short training and class-prediction times (median 0.73 and 0.69 ms, respectively on a 3.5 GHz personal machine). When combined with the MRF-prior, this approach was successfully applied in all eight post-radiotherapy imaging studies and provided visualization and quantification of changes to independent STS sub-regions following radiotherapy for heterogeneous response assessment.

**Interpretation:** Supervised machine-learning approaches to tissue classification in multi-parametric MRI of soft-tissue sarcomas provide quantitative evaluation of heterogeneous tissue changes following radiotherapy.

## Introduction

Soft-tissue sarcoma (STS) is a rare form of cancer that develops in connective tissues. Approximately 3,300 new cases are diagnosed every year in the UK and the 5-years survival rate is ~53% (1). STS tumors are often highly heterogeneous with variable tissue components that include cellular tumor, fat, necrosis, and cystic change. In patients undergoing non-surgical treatments, such as radiotherapy and systemic drug treatments, conventional imaging methods of assessing treatment response are limited as responding tumors may not change in size, or may even grow (pseudoprogression), after treatment (2–4). Hence, more effective and non-invasive methods for assessing treatment response are desired in trials of non-surgical treatments, such as combined radiotherapy with systemic agents. This is particularly difficult since the response of any tumor can be heterogeneous, with different components of a tumor responding differently to the same treatment.

Magnetic resonance imaging (MRI) is widely used in soft-tissue sarcoma, owing to its excellent soft-tissue contrast. Quantitative MRI techniques enable non-invasive investigation of the entire tumor and can provide information about the biological properties of tumors through functional measurements. For example, maps of apparent diffusion coefficient (ADC) derived from diffusion-weighted MRI inform on tissue cellularity, with lower ADC values observed in highly cellular or more aggressive regions within tumor (5). Using contrast enhanced MRI, the time course of T1 signal enhancement after intravenous injection of gadolinium-based contrast agent provides estimates of tumor perfusion and permeability (6). By applying the Dixon MRI techniques, the presence of fat in sarcomas can also be measured and quantified (7).

However, evaluation of multi-parametric quantitative MRI averaged over the entire tumor may not reveal the extent of heterogeneous changes following treatment. By combining quantitative MRI techniques that inform on different aspects of tumor properties (e.g., diffusion-weighted MRI, contrast enhanced MRI and Dixon MRI), it is possible identify sub-components of tumors demonstrating cellular, vascular or fatty phenotypes before and after treatment, thereby enabling tracking and monitoring of the heterogeneity of tumors in response to treatment.

The aim of this pilot study is to evaluate supervised machine learning methods for tissue classification of multi-parametric MRI measurements in soft-tissue sarcomas, and use these methods to quantify post-treatment changes in a cohort of patients treated with radiotherapy.

## Materials and Methods

### Patient Cohort

Eighteen patients with retroperitoneal sarcomas were included in this prospective single-center study (11 male patients and seven female patients; age range 43–76). The study was approved by a national Research Ethics Committee, and all patients gave their written informed consent to participate. Tumors included 14 liposarcomas, two leiomyosarcomas, one spindle cell sarcoma, and one synovial sarcoma. All patients underwent an MRI examination at baseline. In eight patients who were treated with pre-operative radiotherapy (50.4 Gy in 28 fractions) another MRI examination was performed 2–4 weeks after the final fraction of radiotherapy and prior to surgery; 10 patients were treated by surgery alone.

### Imaging Protocol

Patients were scanned on a 1.5 T Siemens MAGNETOM Aera MRI scanner (Siemens Healthcare AG, Erlangen, Germany). Anterior body matrix and posterior spine matrix receive coils were used for image acquisition. Following axial and coronal anatomical T_1_-weighted and T_2_-weighted imaging sequences, functional imaging was performed and consisted of diffusion-weighted imaging (DWI), Dixon imaging, and pre- and post-contrast T_1_-weighted imaging sequences. Images were acquired with a field of view that fully covered the tumor volume; parameters are described in Appendix A and further detailed by Winfield et al. (8) (a second imaging station was used if necessary for large tumors). Post-Gadolinium (Gd) T_1_-weighted images were acquired 4 min after injection of a Gd-based contrast agent (Dotarem, 0.2 ml/kg body weight, administered at 2 ml/s using a power injector).

### Image Analysis

Maps of apparent diffusion coefficient (ADC) were calculated from the DWI and fat-fraction (FF) from Dixon images:

(1) $$\begin{array}{r} \text{FF}=\frac{S_{fat}}{S_{fat}+S_{water}}\times 100\% \end{array}$$

where *S*_*fat*_ and *S*_*water*_ represent the fat and water signals, respectively. Maps of fractional enhancement (EF) were calculated from the pre- and post-Gd T_1_-weighted images using the following equation:

(2) $$\begin{array}{r} \text{EF}=\frac{S_{post}-S_{pre}}{S_{post}+S_{pre}}\times 100\% \end{array}$$

where *S*_*pre*_ and *S*_*post*_ are the signal intensities in pre- and post-Gd T_1_-weighted images, respectively (9). Volumes of interest (VOIs) were defined for each tumor by an expert radiologist with 16 years of experience, outlining the whole tumor on every slice on which the tumor appeared on axial T_2_-weighted images; VOIs were transferred to ADC, FF, and EF maps. All parameter maps were rescaled to ensure values were in the range [0, 1] using the following linear transformations: ADC → ADC/3 × 10^−3^ mm^2^/s, EF → (EF + 100)/200%, and FF → FF/100%. No spatial registration was performed between parameter maps as adequate spatial alignment was verified by a consultant radiologist with experience in STS MRI.

### Tissue Classification

**Figure 1:**
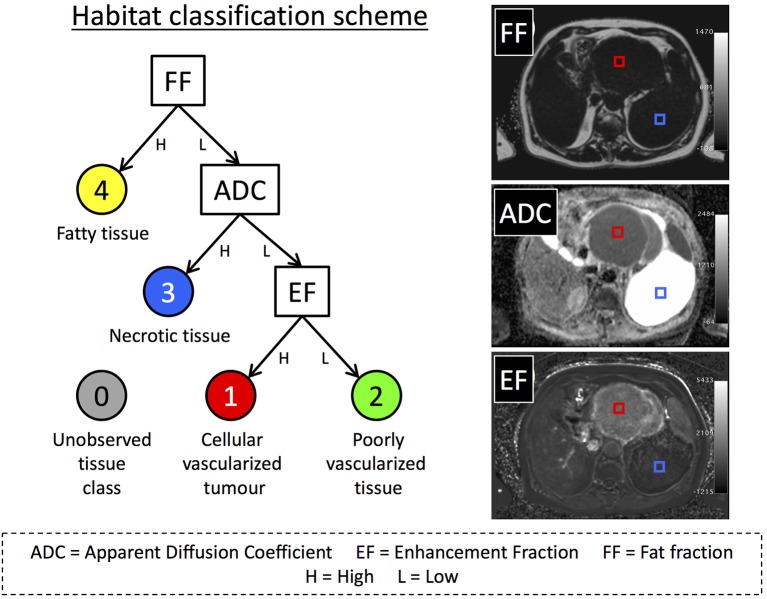
**(Left)** An illustration of our decision tree used to define habitats within our sarcoma population. Classes 3 and 4 were not further divided as cystic/necrotic regions and fat do not enhance in post-Gd images. ADC is not evaluable in fat- suppressed DWI. **(Right)** Images from one patient with a dedifferentiated liposarcoma showing examples of training ROIs positioned in regions corresponding to habitat 1 (red) and habitat 3 (blue). Training ROIs (2 cm^2^) were drawn on either the fat fraction (FF), apparent diffusion-coefficient (ADC), or enhancing fraction (EF) maps, and then transposed onto other maps.

We defined four possible tissue classes for the STS volumes as illustrated in Figure 1, reflecting the aim of segmenting cellular tumor (low ADC, classes 1 and 2) from necrotic/cystic regions (high ADC, class 3), fat (class 4). The cellular tumor was further separated into enhancing (class 1) and non-enhancing (class 2), which may have different biological behavior (2). In addition, we defined a further class to represent the combinations of MRI parameters that were not part of the training data, called “novelties” (10) (class 5). Training data for building the machine-learning classifiers were defined by placing square regions of interest (ROIs) with area 1–2 cm^2^ (45–100 voxels) in regions that exemplified each class, at locations far from visible boundaries (Figure 1). Training ROIs were drawn by a clinical scientist with more than 6 years of experience in tumor analysis and confirmed by a consultant radiologist with 16 years of experience. Between 1 and 4 ROIs were placed in each tumor depending on the classes present, providing a total of 36 ROIs across all 18 patients' baseline scans.

Eights machine-learning (ML) techniques were evaluated for classifying the tissue type for each voxel in this pilot supervised classification exercise using the Scikit-Learn software package (11): Logistic Regression (LR), Support Vector Machine (SVM with a radial basis function), Random Forest (RF), k-Nearest Neighbor (kNN), Kernel Density Estimation (KDE), Naïve-Bayes (NB), and a 20-node, three-layer, fully-connected Neural Network (NN). We also tested a variant of the KDE method where the hyperparameter (bandwidth) was automatically selected using Silverman's approximation (12). To ensure that techniques were sensitive to novelties (voxels that do not represent any of the classes defined in this study), data for an additional 15 ROIs were synthesized by randomly sampling from a uniform distribution covering the intrinsic range of the parameters: EF ∈ [−100, 100] (%), FF ∈ [0, 100] (%), ADC ∈ [0, 3] (×10^−3^ s/mm^2^). All data were normalized to the range [0, 1] prior to training of algorithms. An exhaustive cross-validation approach was used for evaluating classification performance of each of the machine-learning techniques: For each training cycle voxels from one ROI of each class were selected as a validation set, and the ML method under investigation was trained on voxel values from the remaining ROIs. This process was repeated for each unique combination of validation ROIs providing a total of 2,240 training/validation cycles. This process was repeated over the range of hyper-parameters considered for each ML method (see Table 1 for a list of the hyper-parameters considered and their range, along with training/prediction times for each model), and the hyper-parameter that provided the highest median accuracy, defined as the percentage of voxels correctly classified in the validation ROI set, was chosen for further investigation. The data for one patient, for whom 3 different ROI classes had been drawn, was left out of this training/validation phase in order to evaluate the accuracy of these ML methods in an unseen case; this left a total of 33 ROIs for cross-validation analysis. Comparison between methods was achieved using a two-tailed Student's *t*-test (*p* < 0.05 for significance).

**Table 1:** Median training and prediction times for each of the machine-learning techniques used in this study over the range of hyper-parameters tested (5th and 9th percentiles provided in parentheses).

**ML technique**	**Median training time in ms (5th−95th perc.)**	**Median prediction time in ms (5th−95th perc.)**	**Hyper-parameter (range considered for optimization)**
Logistic regression (LR)	9.51 (5.50, 12.58)	0.06 (0.05, 0.11)	**C:** Inverse of the regularization strength **(10**^**−3**^**–10**^**10**^**)**
Support vector machine (SVM)	208.69 (96.01, 1,745.5)	12.94 (5.19, 29.81)	**C:** Penalty parameter that favors smoother decision boundaries when set to a smaller value **(10**^**−3**^**–10**^**5**^**)**
Neural network (NN)	412.23 (47.72, 465.35)	0.24 (0.22, 0.33)	**α:** A L2-regularization parameter that attempts to reduce over-fitting. Smoother decision boundaries with larger values **(10**^**−8**^**–10**^**5**^**)**
Naïve-Bayes (NB)	0.73 (0.70, 1.26)	0.69 (0.66, 1.23)	**None**
Random forest (RF)	399.98 (7.35, 7,304.26)	4.74 (0.23, 86.40)	**N Estimators**: The number of trees being used in the forest **(10–1,000)**
k-Nearest neighbor (kNN)	1.73 (1.64, 2.39)	6.00 (1.20, 67.10)	**N:** The number of closest training data (Euclidean distance) considered to be neighbors of the data being predicted **(10–1,000)**
Kernel density estimation (KDE)	1.57 (1.44, 2.55)	11.33 (6.15, 55.62)	**Bandwidth:** The standard-deviation of the Gaussian kernel used for fitting a KDE model **(10**^**−4**^**–10**^**1**^**)**
Automatic KDE (aKDE)	1.81 (1.54, 2.82)	30.84 (26.83, 36.94)	**None**: Bandwidth is automatically calculated using Silverman's approximation

*Training times are estimated for 1,350–3,000 samples in each case, whilst prediction times are for 135–300 samples (validation step). A brief description of the hyper-parameter used in each case is provided (if applicable), with range test provided in parentheses. Computation times are from a 3.5 GHz personal machine with 16 GB of memory and an Intel Iris Plus graphics card*.

Once the optimum hyper-parameter was selected and models had been trained, they were used to classify the entire tumor volume in all patients, providing a map of the suspected STS tissue sub-type at each voxel location (13) for radiological review. Results were visualized using (i) 3D surface rendering and (ii) color-coded masks overlain on Multi-Planar Reformats (MPRs) of the anatomical images acquired (T2-HASTE). To reduce the level of classification noise observed in the derived habitat maps, a classification de-noising algorithm was used by applying a Markov Random Field (MRF) model to the machine-learned classifications (see Appendix B for the theoretical justification underlying this model, with Python code provided as supplementary file “ml_utilities.py”).

## Results

**Figure 2:**
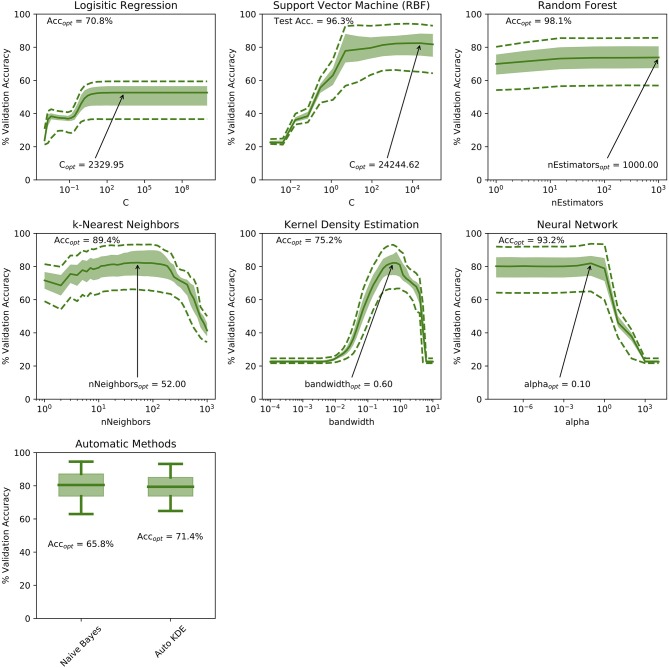
Demonstration of cross-validation accuracies over the range of hyper-parameters tested in this study. For the Kernel Density Estimation and k-Nearest Neighbor methods, an optimum hyper-parameter can be identified. For the remaining techniques, a hyper-parameter limit is identified by the presence of a plateau in the validation accuracy curve. Solid curves represent median values, shaded areas demonstrate the interquartile range and dashed lines represent the 5th and 95th percentiles of the validation accuracy measurements. The optimum hyper parameter is annotated on each sub-plot with the corresponding validation accuracy shown in the top-left.

The cross-validation accuracy for the ranges of hyper-parameters tested in each of the machine-learning methods is demonstrated in Figure 2. For both kNN and KDE methods, optimum hyper-parameters can be established (number of neighbors = 34 and bandwidth = 0.75, respectively). For the remaining ML methods, a plateau is reached in the cross-validation accuracy indicating relative insensitivity to the choice of hyper-parameter after some threshold. Figure 2 also demonstrates the accuracy of each machine learning method on the test ROIs ignored during training: RF classification scored the highest in this case with a test accuracy of 98.1%, and SVM, NN and kNN methods demonstrating slightly lower accuracies of 96.3, 93.2, and 89.4% respectively.

**Figure 3:**
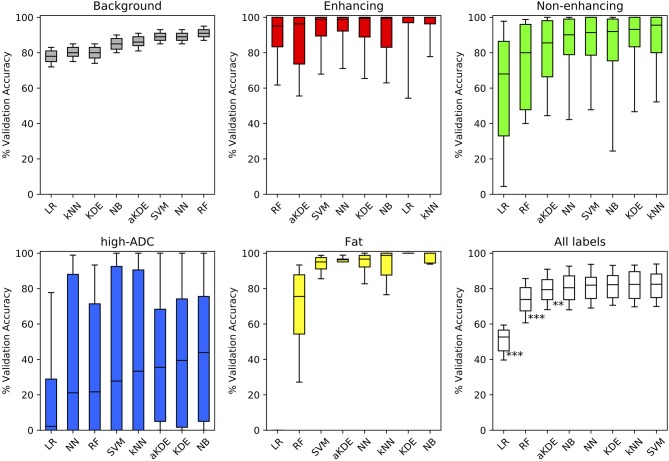
Comparison of the validation accuracy for the different machine learning (ML) techniques applied to our labeled training-set data. Methods are compared for each tissue label separately (colored as per Figure 1) and for all tissue types combined (white). Boxplots demonstrate the distribution of validation accuracies (derived using a randomized cross-validation approach) following optimization of hyper-parameters (bold-lines represent median, shaded areas indicate the inter-quartile range and whiskers the 5th/95th percentiles). Methods are ordered from left to right in order of increasing median accuracy (^**^p < 0.005, ^***^p < 0.0005).

Figure 3 demonstrates the cross-validation accuracy for each of the classes independently and for all classes combined, using the optimal hyper-parameters in each case. The results are sorted in order of ascending median accuracy. NB scored the highest in two out of five tissue classes: (3) high ADC, and (4) fatty tissue, whilst kNN scored highest for discriminating enhancing, well-vascularised (1) from non-enhancing, poorly vascularised (2) tumor tissue. The performance for all tissue types combined demonstrates that in general there is little to choose between 5/8 of these classification methods (NB, NN, KDE, NN, SVM), whilst logistic regression (LR) and random forest classifiers perform poorly in comparison across the tissue sub-types considered. Of the five methods, the Naïve-Bayes (NB) classifier was chosen for further investigation due to its relatively short training and prediction times (Table 1).

**Figure 4:**
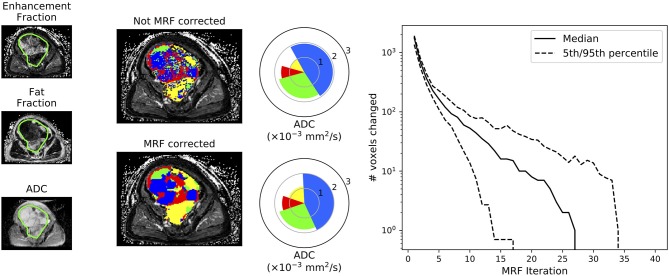
Demonstration of the improvement to tissue sub-region classification following Markov Random Field (MRF) correction of the Naïve-Bayes classifier. This figure demonstrates results for the patient that was not included in the training of our machine-learning approaches (test data). Spie-charts (14) demonstrate the proportion of each tissue sub-compartment within the entire volume as the angle of each segment, whilst the mean ADC of each tissue sub-type is represented by the radius of each segment (note that the ADC of the fat/yellow tissue sub-type from fat-suppressed diffusion-weighted imaging studies should not be interpreted as it will be heavily noise-corrupted; only the proportion/angle of this tissue sub-type is informative). The far-right plot demonstrates the number of voxels that change classification following each iteration through the MRF fitting algorithm across all axial images in this patient: it is evident that the algorithm converges after a finite number of iterations.

Figure 4 compares the classification results of the NB classifier with and without MRF correction on the test-patient that was not included in the initial training of our machine-learning approaches. It is evident that the application of a MRF reduces the classification noise induced when the classifier is applied on a voxel-wise basis without taking into consideration the correlations that are likely to occur between neighboring voxels. This figure also demonstrates the convergent properties of the MRF algorithm, which converged after a median of 27 iterations in this case.

**Figure 5:**
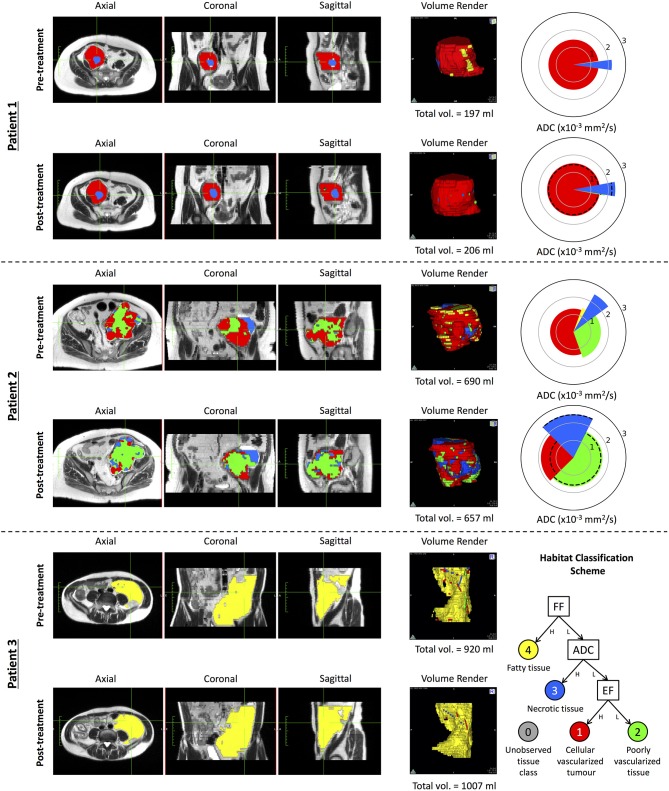
A demonstration of our proposed habitat classification scheme on three patients applied before and after radiation therapy. Spie charts are presented with a radius equal to the mean ADC of the given tissue sub-compartment; dotted lines on the post-treatment Spie charts show the ADC of that tissue sub-type in the pre-treatment data. Multi-planar reformat habitat maps are overlain on T2 HASTE MR-images acquired within the same patient study. **Patient 1** demonstrates a patient with a liposarcoma where a necrotic core is clearly identified (blue) within a majority of strongly enhancing solid tumor (red) prior to treatment. Although there is a marginal increase in the volume of the necrotic core, there little overall change is observed following treatment. **Patient 2** demonstrates data from a pleomorphic sarcoma where there is a clear heterogeneous pattern observed with the majority of the disease consisting of strongly enhancing tumor. Following treatment, there is a marked increase in the proportion of poorly vascularized (green) and necrotic tissue. Within the remaining strongly enhancing tumor after radiotherapy, an increase in mean ADC is observed indicative of treatment response. **Patient 3** demonstrates a well-differentiated liposarcoma with the majority of the tumor consisting of fatty tissue before and after treatment. Results for all eight patients (including these three exemplary patients)with pre-/post-radiotherapy imaging are provided as supplementary information in Appendix C.

We used the NB classifier, in combination with our MRF class-label de-noising algorithm, to investigate the changes occurring to each of the tissue habitats in three patients who received a post-treatment MR exam following radiotherapy (Figure 5). Patient 1 demonstrated STS consisting of mostly viable tumor with high vascularity (class 1 in red), with a necrotic core (class 3 in blue). Following treatment, there was no clear change in the volume of either of these tissue types, nor any change in the ADC (as depicted through a pie-chart in the figure), indicating that the patient did not respond well to treatment. Patient 2 demonstrated with a highly heterogeneous STS, with a mix of tissue classes (1), (2), and (3). Following treatment, there is a clear increase in the proportion of non-enhancing tissue, suggestive of disruption to the vascular supply of the tumor following radiotherapy. When combined with an observed increase in ADC for the remaining well-vascularized tissue, this may provide evidence of tumor response to radiotherapy, regardless of the absence of any significant change in tumor volume (5.7% reduction following treatment). Patient 3, however, demonstrated highly fatty, well-differentiated liposarcoma, which has been well-described through our approach; no change is found following radiotherapy. Results for all eight patients are provided in Appendix C.

## Discussion

Soft-tissue sarcoma is a highly heterogeneous disease, and there remains a lack of appropriate imaging biomarkers for monitoring the success of therapy. Novel therapeutic agents or radiotherapy may not result in a significant change in tumor size, but in a heterogeneous change in the tumor composition. In this technical development study, we have investigated the use of a number of machine-learning approaches for automatically segmenting the heterogeneous tissue compartments within STS, thereby providing a map that aims to characterize the tumor microenvironment for radiological review. This approach facilitates the quantification of changes in ADC, fat-fraction and enhancement-fraction estimated through co-registered, multi-parametric MRI occurring in each of the segmented tissue classes, and may provide a novel response biomarker in STS.

Out of the eight machine-learning approaches we investigated, we found that 5/8 methods did not outperform each other in terms of segmentation accuracy. This is likely due to the fact that our data is intrinsically low-dimensional (only three parameters per-voxel: ADC, enhancement-fraction and fat-fraction), and most of the techniques provide enough degrees of freedom to account for the variation of these parameters for the different classes investigated for STS. This is supported by the relatively poor performance of logistic regression, which was unable to model the full complexity of the data space.

In addition, we have investigated inclusion of the estimated class probabilities from machine-learning classification methods into a Markov Random Field framework, which allows for de-noising of the estimated habitat maps by introducing a spatial prior distribution on the segmented regions. This technique provided smoother classification maps when compared to classification based purely on the trained ML architectures alone. This approach could well be extended to any other machine-learning task where the classifications of a group of input data are not expected to be independent (15–17).

Although previous authors have investigated the role of machine learning for the segmentation of sarcoma using MRI data, these reports focused on the utility of dynamic contrast-enhanced MRI alone, and did not exploit the multi-parametric capabilities of MRI for determining a more complete habitat image of the tumor, as explored here (18, 19). Moving forward, there is a clear need to explore a larger patient population for further validation of the methods described in this article. This should include multi-center studies to determine the sensitivity of the technique to images acquired from multiple vendors and at different institutions (20). Another important consideration is when MRI studies should be performed following neoadjuvant radiotherapy in order to observe a measureable treatment-induced change; the effects of treatment may not manifest immediately after the final radiation dose. However, the timing of imaging after neoadjuvant radiotherapy is limited by surgery, which is typically performed at 4–6 weeks post-treatment. Imaging following radiotherapy to non-resectable disease may enable insight into later effects. The segmentation methodology would also benefit from repeatability testing to determine its sensitivity as a radiotherapy response biomarker (21). A limitation of this study is that one expert radiologist generated training data samples in the patients investigated, and so further work may investigate the user-repeatability for generating gold-standard training data. The regions chosen for training data would ideally be validated through post-operative histopathological confirmation of the tissue type in that region. There may also be scope for including more complex deep-learning approaches for producing habitat maps for soft-tissue sarcoma, including methods, such as U-Net convolutional networks (22), but these techniques would require a much larger cohort size, which may be unfeasible in a population with a rare cancer type. Lastly, the cohort of eight patients who received radiotherapy had STS tumors that were predominantly well-vascularized, and future randomized studies should include patients with more heterogeneous tumor phenotypes. However, the full cohort of this study, which included patients for whom no radiotherapy was delivered, provided sufficient examples of each tissue class to evaluate this technological development.

Modern advances in artificial intelligence and machine-learning are anticipated to improve automatic segmentation accuracies in the next few years, and supersede conventional image-processing methods for extracting regions of interest in medical imaging datasets. We have demonstrated that a variety of simple machine-learning approaches can be used to automatically extract sub-regions in a highly heterogeneous tumor phenotype, and that quantification of the volume and ADC within these regions may provide a radiotherapy response biomarker in soft-tissue sarcoma. Tools, such as these will facilitate clinical decision making for a disease that can be difficult to manage, and thus may promote personalized treatment regimens and improve patient outcome. Intra-tumoural heterogeneity confounds the interpretation of treatment response in many other, more common cancers; provided sufficient data is acquired, we envisage that these methods will be highly applicable in many prospective cancer studies investigating tumor response to targeted therapeutics.

## Data Availability Statement

Datasets are available upon request from the authors. Code developed is provided in Appendix D.

## Ethics Statement

This study was carried out in accordance with the Declaration of Helsinki (1996) and conditions of the ethical approval obtained from the National Research Ethics Service (NRES) committee Cambridge East REC: 13/EE/1086.

## Author Contributions

MB, JW, AM, DS, KT, VM, DC, D-MK, ML, and CM: literature search. MB, JW, and CM: figures. MB, JW, AM, DS, KT, DC, and CM: study design. JW, AM, DS, KT, VM, DC, and CM: data acquisition. MB, JW, DC, and CM: data analysis. MB, JW, DC, D-MK, and CM: data interpretation. MB and JW: software development. MB, JW, DC, D-MK, ML, and CM: article writing.

### Conflict of Interest

The authors declare that the research was conducted in the absence of any commercial or financial relationships that could be construed as a potential conflict of interest.
