# Supplementary material for: Supervised Machine-Learning Enables Segmentation and Evaluation of Heterogeneous Post-treatment Changes in Multi-Parametric MRI of Soft-Tissue Sarcoma
Source: Front Oncol. 2019 Oct 10;9:941. doi: 10.3389/fonc.2019.00941 (PMC6795696; doi:10.3389/fonc.2019.00941)
Supplement: Supplementary file 1 [file Data_Sheet_1.PDF]

Appendix A. MR-imaging parameters

	DWI	Dixon	Pre- and post-Gd T_1-weighted imaging
**Sequence**	ss-EPI	3D FLASH	3D FLASH
**Slice orientation**	Axial	Axial	Axial
**PE direction**	AP	AP (in-plane)	AP (in-plane)
**FOV / mm x mm**	420 x 336	380 x 285	380 x 285
**Acquired matrix**	160 x 128	256 x 144	256 x 144
**Slice thickness / mm**	5	5	5
**Slice gap**	0	0	0
**Number of slices**	40 slices per station (>1 station if necessary to cover tumour)	40 slices per slab (>1 station if necessary to cover tumour)	40 slices per slab (>1 station if necessary to cover tumour)
**Echo time (TE) / ms**	65	2.39 and 4.77	1.1
**Repetition time (TR) / ms**	9200	7.05	3.8
**Nutation angle / °**	90	3	17
**Receiver bandwidth / Hz/pixel**	1955	400	250
**Number of signal averages (NSA)**	NSA = 4 for b = 50 s mm^{-2} and b = 600 s mm^{-2}; NSA = 5 for b = 900 s mm^{-2}	1	1
**Reduced acquisition**	GRAPPA, reduction factor 2	CAIPIRINHA, acceleration factor 2x2	None
**Fat suppression**	SPAIR	None	None
**Breathing instructions**	Free breathing	Breath-hold on expiration	Breath-hold on expiration
**Diffusion weightings / s mm^{-2}**	50, 600, 900	n/a	n/a
**Diffusion encoding scheme**	Three-scan trace	n/a	n/a
**Acquisition time**	6.5 minutes per station (2 stations if required)	14 seconds per station (2 stations if required)	22 seconds per imaging volume (acquired pre- and post-contrast injection, with 2 stations if required)

	Axial T1w imaging	Coronal T1w imaging	Axial T2w imaging	Coronal T2w imaging
**Sequence**	2D FLASH	2D FLASH	HASTE	HASTE
**Slice orientation**	Axial	Coronal	Axial	Coronal
**PE direction**	AP (in-plane)	LR (in-plane)	AP (in-plane)	LR (in-plane)
**FOV / mm x mm**	380 x 285	345 x 380	380 x 285	345 x 380
**Acquired matrix**	256 x 192	290 x 320	320 x 240	290 x 320
**Slice thickness / mm**	5	7	5	5
**Slice gap**	0	0	0	0
**Number of slices**	40 slices per slab (>1 station if necessary to cover tumour)	28 slices per slab	40 slices per slab (>1 station if necessary to cover tumour)	34 slices per slab
**Echo time (TE) / ms**	4.86	4.86	88	88
**Repetition time (TR) / ms**	371	97	1000	1000
**Nutation angle / °**	70	70	170	1000
**Receiver bandwidth / Hz/pixel**	230	230	475	475
**Number of signal averages (NSA)**	1	1	1	1
**Reduced acquisition**	GRAPPA, reduction factor 2	GRAPPA, reduction factor 2	GRAPPA, reduction factor 2	GRAPPA, reduction factor 2
**Fat suppression**	None	None	None	None
**Breathing instructions**	Breath-hold on expiration (2 concatenations)	Breath-hold on expiration (3 concatenations)	Breath-hold on expiration (2 concatenations)	Breath-hold on expiration (2 concatenations)
**Diffusion weightings / s mm^{-2}**	n/a	n/a	n/a	n/a
**Diffusion encoding scheme**	n/a	n/a	n/a	n/a
**Acquisition time**	26 seconds/ concatenation	16 seconds/ concatenation	21 seconds/ concatenation	18 seconds/ concatenation
