# Supplementary material for: Supervised Machine-Learning Enables Segmentation and Evaluation of Heterogeneous Post-treatment Changes in Multi-Parametric MRI of Soft-Tissue Sarcoma
Source: Front Oncol. 2019 Oct 10;9:941. doi: 10.3389/fonc.2019.00941 (PMC6795696; doi:10.3389/fonc.2019.00941)
Supplement: Supplementary file 2 [file Data_Sheet_2.PDF]

# Appendix B. On the utility of Markov Random Fields for machine-learning based classifications.

## 1 Markov Random Fields

In this section we present a brief overview of the theory of Markov Random Fields (MRF) applied to classification problems. For a more complete treatise on the subject the reader is referred to previous texts, including seminal works by Geman and Geman [1], and Besag [2, 3], from whom much of the notation in the text below is borrowed.

Consider the classification problem as being one of determining a label $x \in \mathcal{L} = \{L_1, L_2, \ldots, L_M\}$ from $M$ possible labels, for some input recorded data $y$. One approach to this is to construct a probability model for the label given the input data from Bayes' theorem:

$$p(x = L_m|y) = \frac{p(y|x = L_m)p(x = L_m)}{\sum\limits_{x \in \mathcal{L}} p(y|x = L_m)p(x = L_m)}$$

where $p(y|x)$ is *likelihood* of the data given the label and $p(x)$ is the *prior probability* of the given label. The label is then chosen that maximises this *a-posteriori* probability distribution:

$$\hat{L} = \arg\max_{L \in \mathcal{L}} p(x = L|y)$$

In many applications it is required to perform many such classifications for multiple data values $y_i$, where $i \in \{1, 2, \ldots, N\} = \mathcal{S}$ is the index of each datum within the scene, $\mathcal{S}$. An example of such a problem occurs in medical imaging, where $y_i$ represents a noisy pixel value at position $i$ obtained from a medical imaging device, and $x_i$ is the tissue type at that position; the distribution of the image noise is captured through the likelihood function of the model. Image segmentation then becomes a matter of determining the full set of labels $\mathbf{x} = \{x_1, x_2, \ldots, x_N\}$ from the image pixel intensities $\mathbf{y} = \{y_1, y_2, \ldots, y_N\}$ (note that $y_i$ maybe vector valued if, for example, multiple imaging modalities have been used). The segmentation problem is then to determine the complete set of labels $\mathbf{x}$ given

all data $\mathbf{y}$:

$$\begin{aligned}\hat{\mathbf{x}} &= \arg\max_{\mathbf{x}} p(\mathbf{x}|\mathbf{y}) \\ &= \arg\max_{\mathbf{x}} \frac{p(\mathbf{y}|\mathbf{x})p(\mathbf{x})}{\sum_{\mathbf{x}\in\chi} p(\mathbf{y}|\mathbf{x})p(\mathbf{x})} = \arg\max_{\mathbf{x}} \frac{1}{Z} p(\mathbf{y}|\mathbf{x})p(\mathbf{x})\end{aligned} \tag{1}$$

where $\chi$ represents the space of all $M^N$ possible configurations of $\mathbf{x}$. We make the assumption that recorded data values are conditionally independent given their labels, and that the likelihood function for each datum is identical (i.e. the noise distribution is constant):

$$p(\mathbf{y}|\mathbf{x}) = \prod_{i=1}^{N} p(y_i|x_i)$$

In general, the prior distribution of the full set of labels may not be conditionally independent, but is better described as a Markov Random Field (MRF): in true segmentation problems pixels within a small region of the image are more likely to have the same label (liver, kidneys, brain etc.). Unfortunately, this makes direct calculation of equation 1 difficult as the normalisation constant $Z$ cannot be determined.

The underlying property of a MRF is that

$$p(x_i|\mathbf{x}_{\mathcal{S}\backslash i}) = p(x_i|\mathbf{x}_{\partial i}) \tag{2}$$

that is, the conditional probability of label $x_i$ given all other labels, $x_{\mathcal{S}\backslash i}$, is equal to the conditional probability of the label given only the labels within the local *neighborhood* of $i$, $\partial i$. As discussed by Besag [3], this imposes a constraint on the functional form of $p(\mathbf{x})$ (the Hammersley-Clifford Theorem):

$$\begin{aligned} p(\mathbf{x}) \propto \exp\Bigg\{ &\sum_{i=1}^{N} \alpha_i(x_i) + \sum_{i=1}^{j-1}\sum_{j=2}^{N} \beta_{i,j}(x_i, x_j) + \\ &\sum_{i=1}^{j-1}\sum_{j=2}^{k-1}\sum_{k=3}^{N} \gamma_{i,j,k}(x_i, x_j, x_k) + \cdots + \omega_{1,2,\ldots,N}(x_1, x_2, \ldots, x_N) \Bigg\} \end{aligned} \tag{3}$$

In this notation, the functions $\alpha$, $\beta$, and $\gamma$ represent the first, second and third-order (and so on) interactions between labels, and no restrictions are placed on their form; in many image-processing applications, only second order MRF's are of interest and so only $\alpha$ and $\beta$ are non-zero (without loss of generality we shall assume this from now on). The conditional probability of labels given the neighbourhood is then found to be:

$$p(x_i|\mathbf{x}_{\mathcal{S}\backslash i}) = \frac{p(\mathbf{x})}{p(\mathbf{x}_{\mathcal{S}\backslash i})} = \frac{1}{Z'} \exp\left\{ \alpha_i(x_i) + \sum_{j\neq i} \beta_{i,j}(x_i, x_j) \right\} \tag{4}$$

where $p(x_i|\mathbf{x}_{\partial i}) = p(x_i|\mathbf{x}_{\mathcal{S}\backslash i})$ as long as $\beta_{i,j}(\cdot) = 0$ if $j \notin \partial i$. In this case the normalisation constant $Z'$ can be calculated directly by summing the exponential over all possible labels.

An example of a simple MRF used in image segmentation is the Ising model. For a 2-dimensional image, the neighbourhood is set to consist of the 4 pixels above (N), below (S), left (W) and right (E) of pixel $i$. We set $\alpha = 0$ (no label is believed to be more prevalent than the others), and $\beta$ as:

$$\beta_{i,j}(x_i, x_j) = b \cdot I(x_i, x_j)$$
$$I(x_i, x_j) = \begin{cases} 1, & x_i = x_j \\ 0, & x_i \neq x_j \end{cases}$$

When $b > 0$, this indicates that neighbouring pixels are likely to share the same label. In the experiments performed in this article we chose to use such an Ising model with $b = 1.5$.

There are many proposed methods for solving equation 1 given the form of Markov Random Fields discussed above. These include (i) simulated annealing through a Gibbs sampling approach [1], (ii) iterated conditional modes (ICM) where the label of each pixel is chosen to maximise the probability of the label given the data and neighbouring labels [2], and (iii) loopy belief propagation [4] to name a few. The advantage to all of these approaches is that they iteratively solve the segmentation problem for each pixel in turn, using the input noisy data and the prior probability of the label given the labels of the neighbouring pixels (equation 4). This is a much simpler problem to solve, and many authors have proven either equivalence, or at least good approximation to determining the complete solution (equation 1).

# 2 Application of Markov random fields to classification systems derived from machine-learning approaches

Machine learning (ML) techniques are now demonstrating remarkable classification accuracy when compared with conventional approaches. ML allows for a completely data-centric approach to classification without the requirement for proposing a likelihood model or a prior probability model for the classification task. From an adequate number of training samples (paired input data $\mathbf{y}$ with ground-truth classes $\mathbf{x}$), ML attempts to determine an optimal classifier, $f : Y \to X$ to accurately classify future samples for which the underlying class is unknown. Through approaches such as Platt scaling [5] and the use of the softmax output layer for Neural Networks, it is also possible to determine the probability of a class given the input data for any ML classification method (including those that are not intrinsically probabilistic). From a general perspective, the

probabilities generated by these approaches may be modelled as

$$\begin{aligned} p_{ML}(x_i|y_i) &= \frac{p(y_i|x_i)p(x_i)}{\sum\limits_{x_i} p(y_i|x_i)p(x_i)} \\ &= \frac{1}{W} p(y_i|x_i)p(x_i) \end{aligned} \tag{5}$$

where $p(y_i|x_i)$ is some unknown likelihood function and $p(x_i)$ is an unknown prior probability (as illustrated in Figure 1).

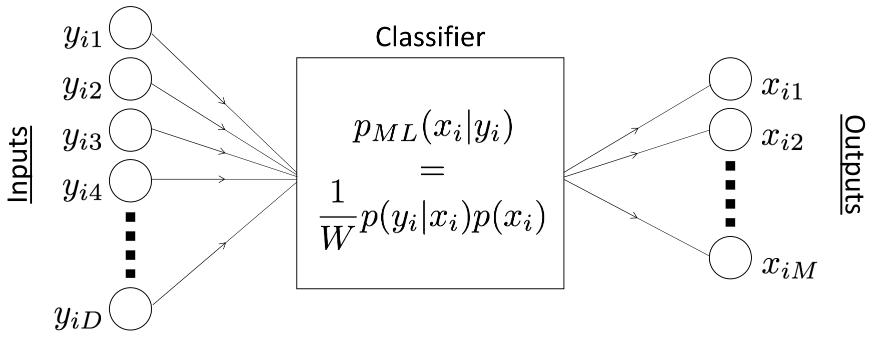


Figure 1: Basic illustration of the machine learning classifier.

An important distinction between this model and the models discussed above for MRFs, is that ML classifiers consider each set of input data as independent, and so we must also assume that the a-posteriori classification probabilities provided are conditionally independent:

$$p_{ML}(\mathbf{x}|\mathbf{y}) = \prod_{i=1}^{N} p_{ML}(x_i|y_i)$$

In many applications (including image segmentation) it would advantageous to impose a MRF prior to the output probabilities from ML classification methods, but unfortunately this is not possible to do directly. In MRF classification problems, we require the conditional a-posterior distribution for each pixel location, $i$:

$$p_{MRF}(x_i|y_i, x_{\partial i}) = \frac{1}{Z} \cdot p(y_i|x_i) \cdot \exp\{\alpha_i(x_i)\} \cdot \exp\left\{ \sum_{j \in \partial i} \beta_{i,j}(x_i, x_j) + \dots \right\} \tag{6}$$

In the above, the prior has been separated into two terms: one that is independent of the pixel neighbours (the $\alpha$ function) and one that is not ($\beta$ function and any higher-order terms if present). Comparing this with equation 5, and assuming the likelihood functions are equivalent, it is possible to attribute the prior determined by the ML classifier, $p(x_i)$, to the $\alpha$ functional of the MRF prior. For MRF inference methods

such as iterated conditional modes (ICM), where the aim is to determine the only the maximum *a-posteriori* label, it is also possible to incorporate the second-order terms of the MRF prior into the ML model. Maximising the posterior probability in equation 6 with respect to $x_i$ is equivalent to maximising the natural logarithm of the posterior in equation 5 with an additional term indicating the dependencies between neighbouring pixel labels:

$$\begin{aligned}
&\arg\max_{x_i} \{p_{MRF}(x_i|y_i, x_{\partial i})\} \\
&\qquad = \arg\max_{x_i} \{\ln [p_{MRF}(x_i|y_i, x_{\partial i})]\} \\
&\qquad = \arg\max_{x_i} \left\{ \ln [p(y_i|x_i)] - \ln Z + \alpha_i(x_i) + \sum_{j \in \partial i} \beta_{i,j}(x_i, x_j) + \dots \right\} \\
&\qquad = \arg\max_{x_i} \left\{ \ln [p(y_i|x_i)] - \ln W + \ln [p(x_i)] + \alpha_i^*(x_i) + \sum_{j \in \partial i} \beta_{i,j}(x_i, x_j) + \dots \right\} \\
&\qquad = \arg\max_{x_i} \left\{ \ln [p_{ML}(x_i|y_i)] + \alpha_i^*(x_i) + \sum_{j \in \partial i} \beta_{i,j}(x_i, x_j) + \dots \right\} \qquad (7)
\end{aligned}$$

where we have explicitly set $\alpha_i(x_i) = \ln [p(x_i)] + \alpha_i^*(x_i)$ by choice ($\alpha^*$ provides an additional arbitrary prior on $x_i$ if required, but can be set to zero). The terms $\ln W$ and $\ln Z$ maybe removed and/or included in the above as they are independent of $x_i$ and thus do not modify the maximisation problem.

Using this result we use the following variant of the ICM algorithm, which utilises posterior-probabilities derived from an arbitrary machine-learning classifier:

### Inputs

- An image of pixel intensities $\mathbf{y}$
- A machine-learning classification model that has previously been trained using appropriate data
- The relevant hyper-parameters for the chosen MRF model (i.e. the functional form of $\beta_{i,j}(\cdot)$ and any higher order terms)

### Outputs

- An estimate of the labels within the image, $\hat{\mathbf{x}}$

**Algorithm**

1. Produce an initial estimate of $p^0_{ML}(x_i|y_i)$ for each pixel location $i \in \mathcal{S}$.
2. Maximise $p^0_{ML}(x_i|y_i)$ at each pixel location to produce an initial estaimte of the labels $\hat{\mathbf{x}}^0$
3. For each pixel $i \in \mathcal{S}$, compute the pseudo log-posterior probability as

$$\ln\left[p^t_{MRF}(x_i|y_i\right] = \ln\left[p^{t-1}_{ML}(x_i|x_i)\right] + \sum_{j\in\partial i}\beta_{i,j}(x_i, y_j) + \dots$$

4. Maximise the pseudo log-posterior probability for each pixel location $i \in \mathcal{S}$ to produce an updated estimate of the labels $\hat{\mathbf{x}}^t$
5. Optional. Use the labels $\hat{\mathbf{x}}^t$ and the image $\mathbf{y}$ to re-train the machine-learning model and determine updated values for $p^t_{ML}(x_i|y_i)$
6. If $|\hat{\mathbf{x}}^t - \hat{\mathbf{x}}^{t-1}| \leq \varepsilon$ then break the loop and return $\hat{\mathbf{x}}^t$, else return to step (3)

where $\varepsilon$ is some acceptable level of tolerance used to check for convergence.

# References


[1] S Geman and D Geman. Stochastic relaxation, gibbs distributions, and the bayesian restoration of images. *IEEE Trans Pattern Anal Mach Intell*, 6(6):721–41, Jun 1984.

[2] Julian Besag. On the statistical analysis of dirty pictures. *Journal of the Royal Statistical Society. Series B (Methodological)*, pages 259–302, 1986.

[3] Julian Besag. Spatial interaction and the statistical analysis of lattice systems. *Journal of the Royal Statistical Society. Series B (Methodological)*, 36(2):192–236, 1974.

[4] Judea Pearl. *Reverend Bayes on inference engines: A distributed hierarchical approach.* Cognitive Systems Laboratory, School of Engineering and Applied Science, University of California, Los Angeles, 1982.

[5] John Platt et al. Probabilistic outputs for support vector machines and comparisons to regularized likelihood methods. *Advances in large margin classifiers*, 10(3):61–74, 1999.
