# Supplementary material for: Supervised Machine-Learning Enables Segmentation and Evaluation of Heterogeneous Post-treatment Changes in Multi-Parametric MRI of Soft-Tissue Sarcoma
Source: Front Oncol. 2019 Oct 10;9:941. doi: 10.3389/fonc.2019.00941 (PMC6795696; doi:10.3389/fonc.2019.00941)
Supplement: Supplementary file 3 [file Data_Sheet_3.PDF]

Appendix C: All images within this Appendix are displayed in the same format; the five other patients demonstrate little change following radiotherapy where the entire tumor volume falls into category 1 before and after treatment.

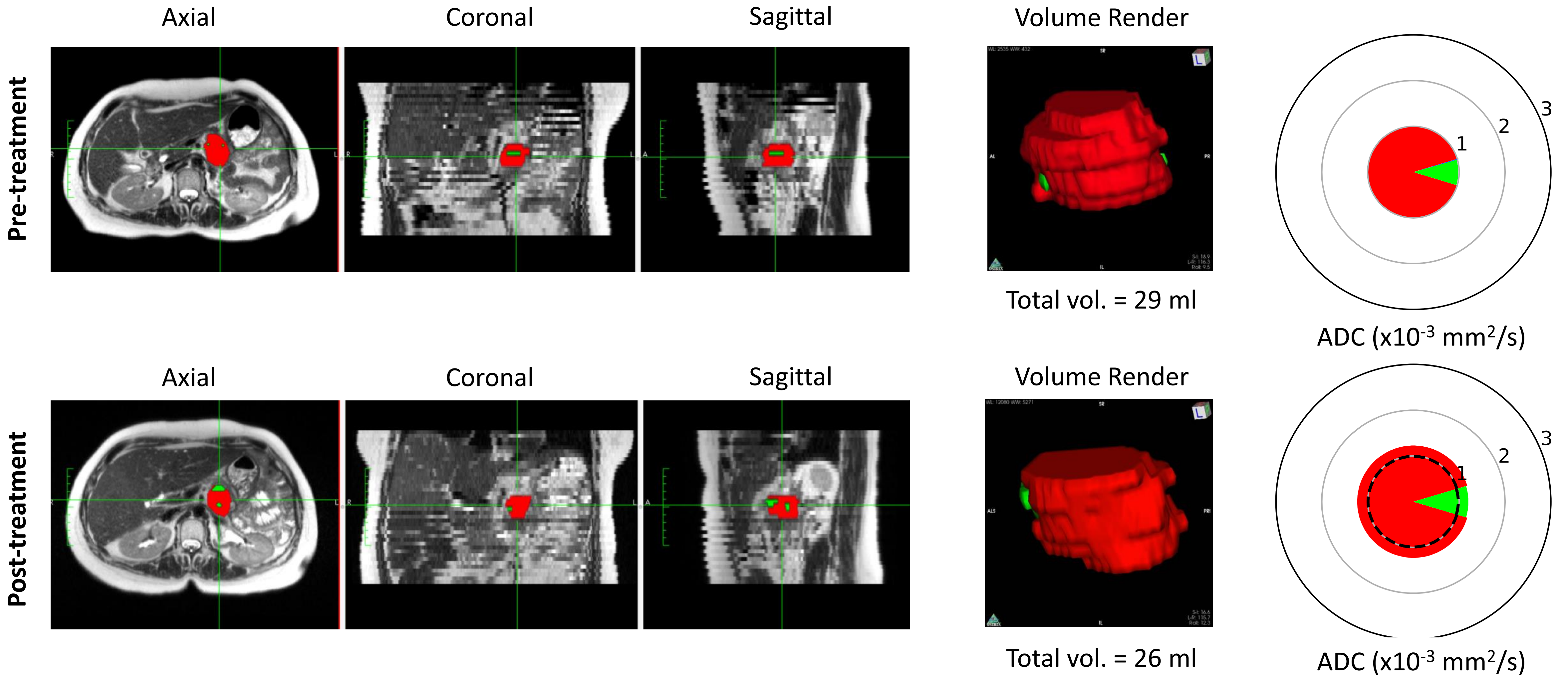
Axial
Coronal
Sagittal
Volume Render
Pre-treatment
Total vol. = 29 ml
1
2
3
ADC (x10-3 mm2/s)
Axial
Coronal
Sagittal
Volume Render
Post-treatment
Total vol. = 26 ml
1
2
3
ADC (x10-3 mm2/s)

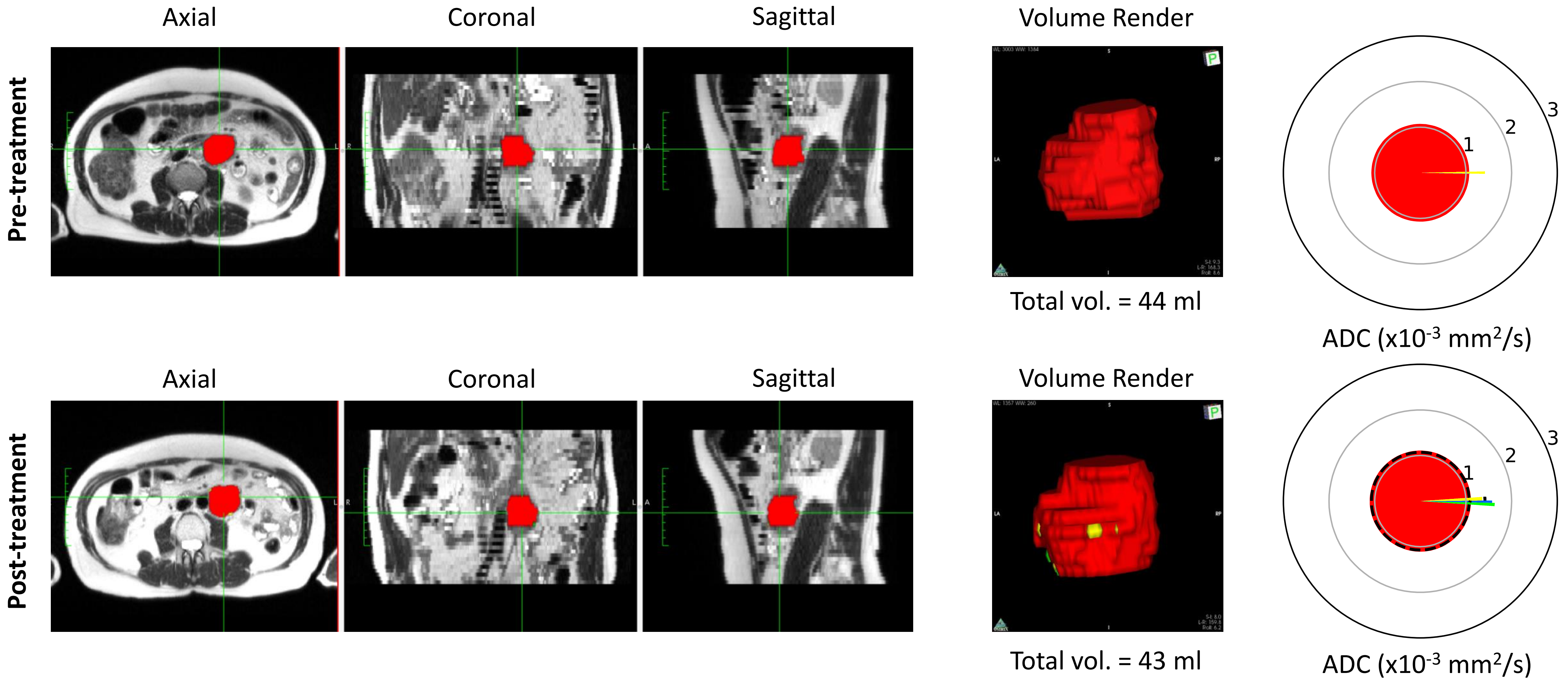
Axial
Coronal
Sagittal
Volume Render
Pre-treatment
Total vol. = 44 ml
1
2
3
ADC (x10-3 mm2/s)
Axial
Coronal
Sagittal
Volume Render
Post-treatment
Total vol. = 43 ml
1
2
3
ADC (x10-3 mm2/s)

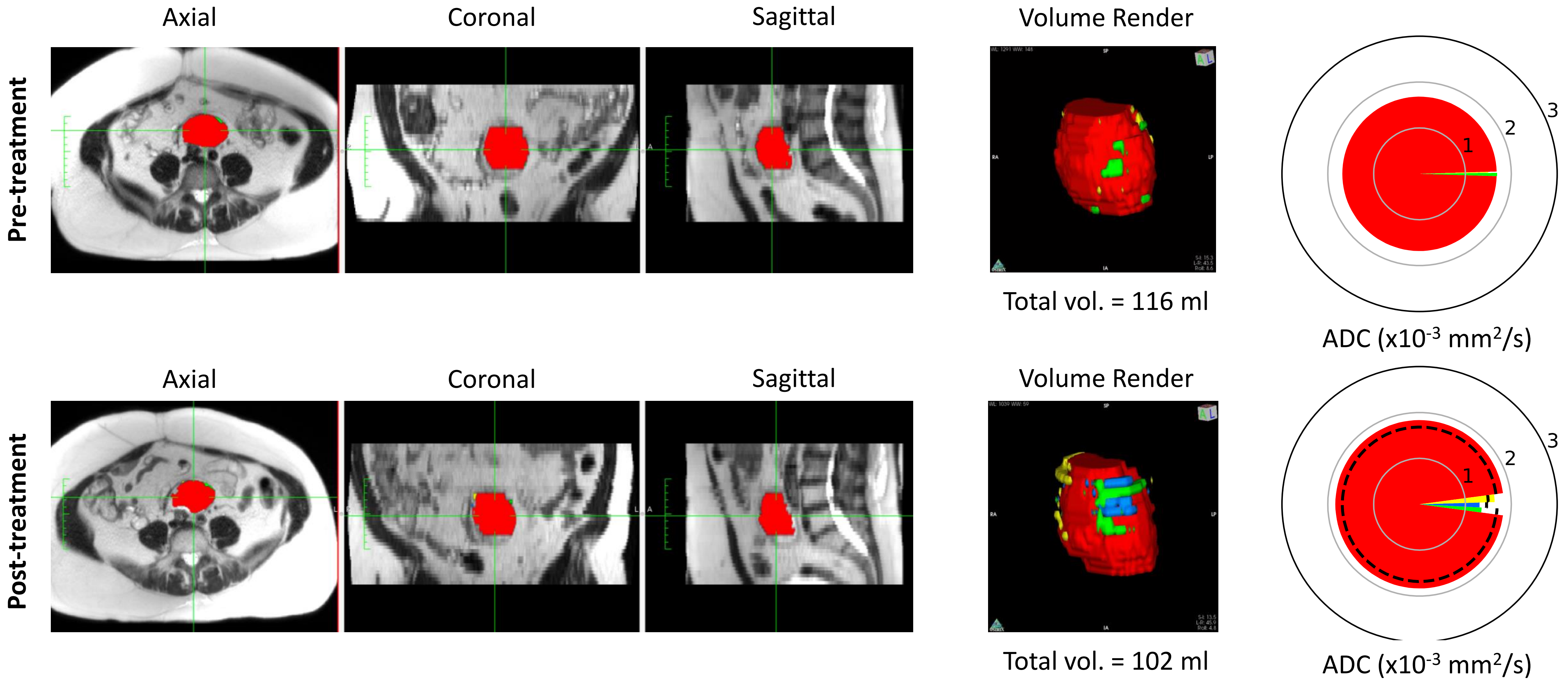
Axial
Coronal
Sagittal
Volume Render
Pre-treatment
Total vol. = 116 ml
1
2
3
ADC (x10-3 mm2/s)
Axial
Coronal
Sagittal
Volume Render
Post-treatment
Total vol. = 102 ml
1
2
3
ADC (x10-3 mm2/s)

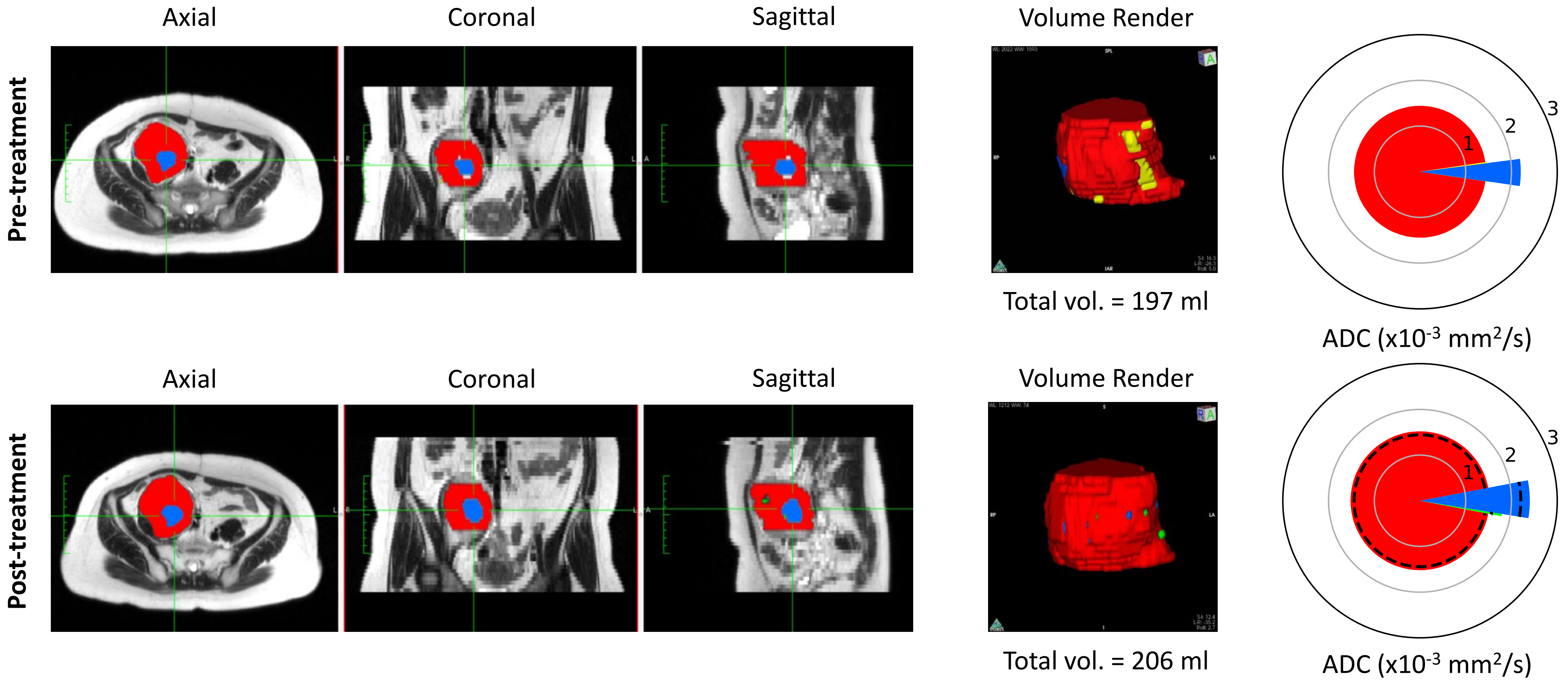
Axial
Coronal
Sagittal
Volume Render
Pre-treatment
Total vol. = 197 ml
1
2
3
ADC (x10-3 mm2/s)
Axial
Coronal
Sagittal
Volume Render
Post-treatment
Total vol. = 206 ml
1
2
3
ADC (x10-3 mm2/s)

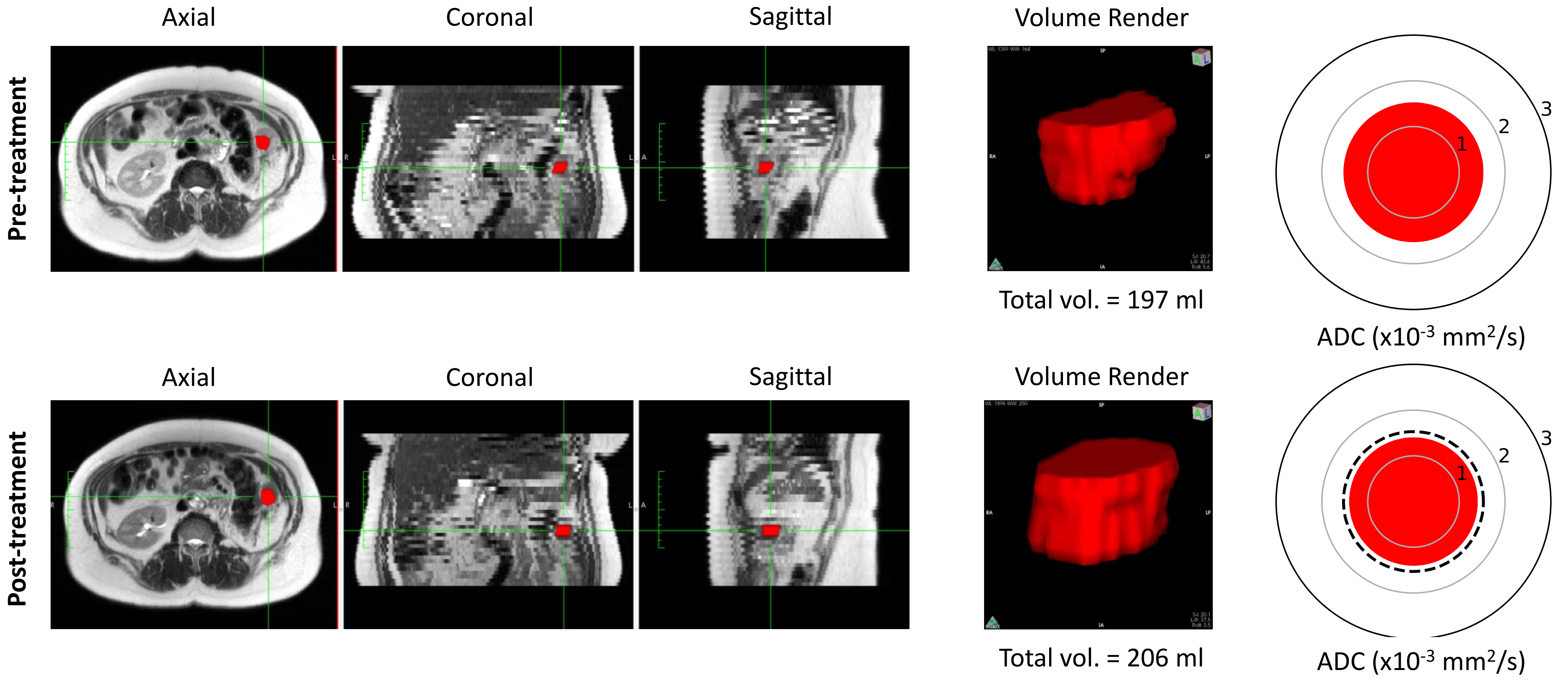
Axial
Coronal
Sagittal
Volume Render
Pre-treatment
Total vol. = 197 ml
1
2
3
ADC (x10^{-3} mm^2/s)
Axial
Coronal
Sagittal
Volume Render
Post-treatment
Total vol. = 206 ml
1
2
3
ADC (x10^{-3} mm^2/s)

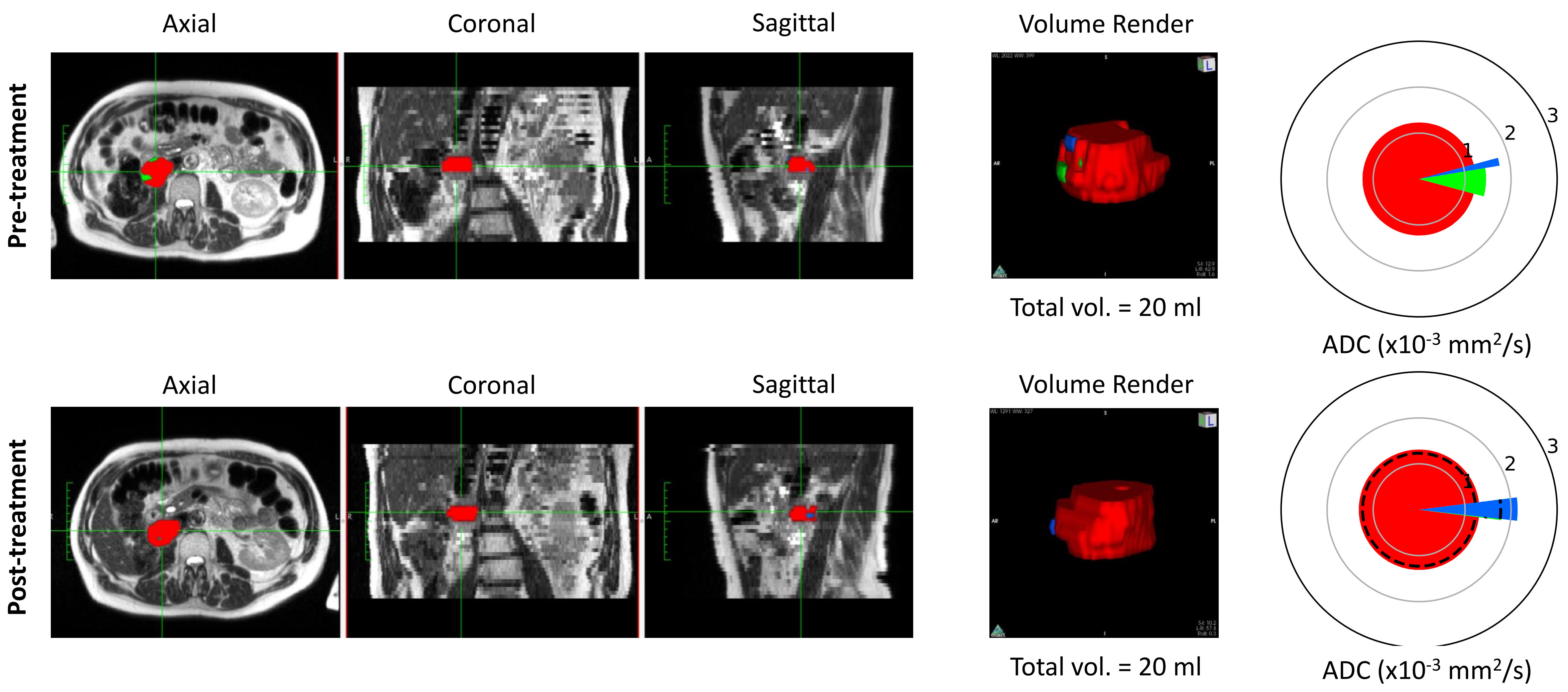
Pre-treatment
Axial
Coronal
Sagittal
Volume Render
Total vol. = 20 ml
ADC (x10^{-3} mm^2/s)
1
2
3
Post-treatment
Axial
Coronal
Sagittal
Volume Render
Total vol. = 20 ml
ADC (x10^{-3} mm^2/s)
1
2
3

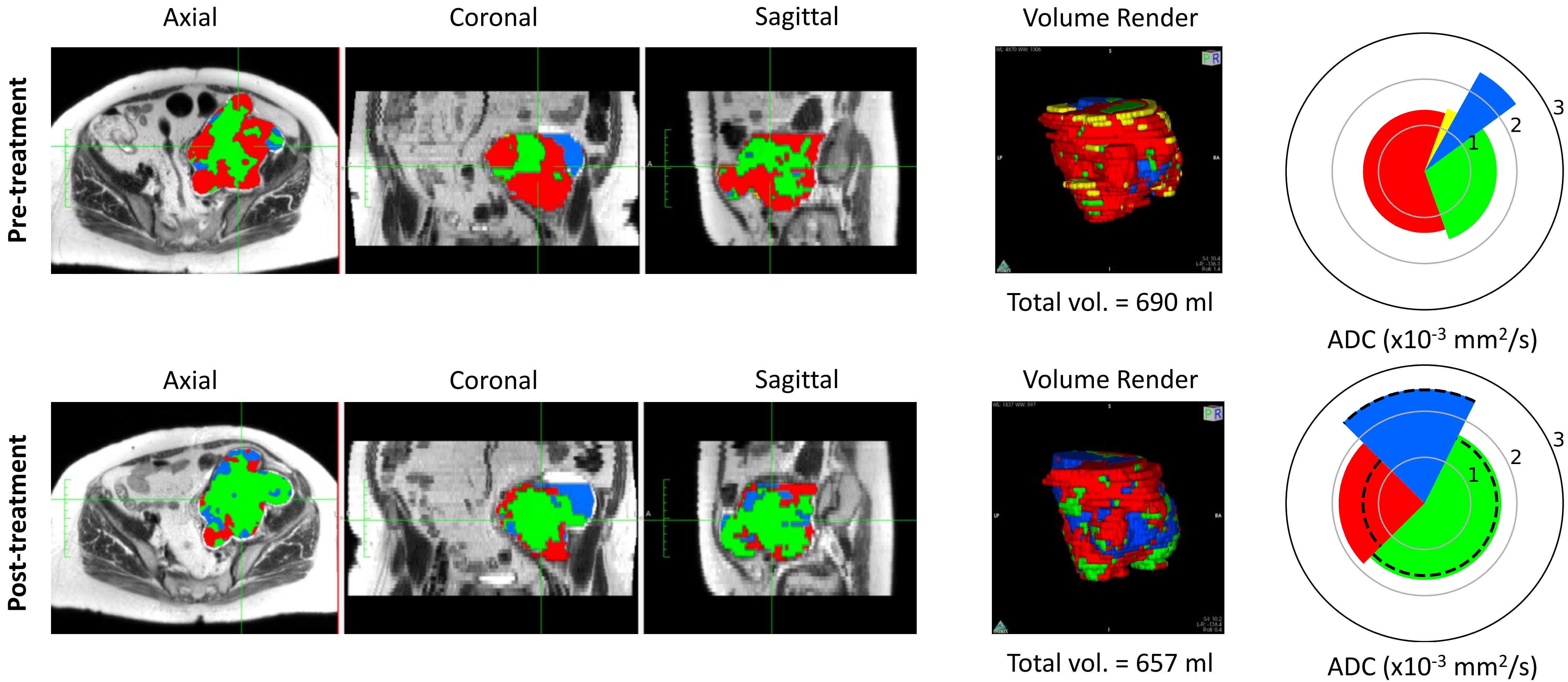

Axial
Coronal
Sagittal
Volume Render
Pre-treatment
Total vol. = 690 ml
ADC (x10-3 mm2/s)
1
2
3
Axial
Coronal
Sagittal
Volume Render
Post-treatment
Total vol. = 657 ml
ADC (x10-3 mm2/s)
1
2
3

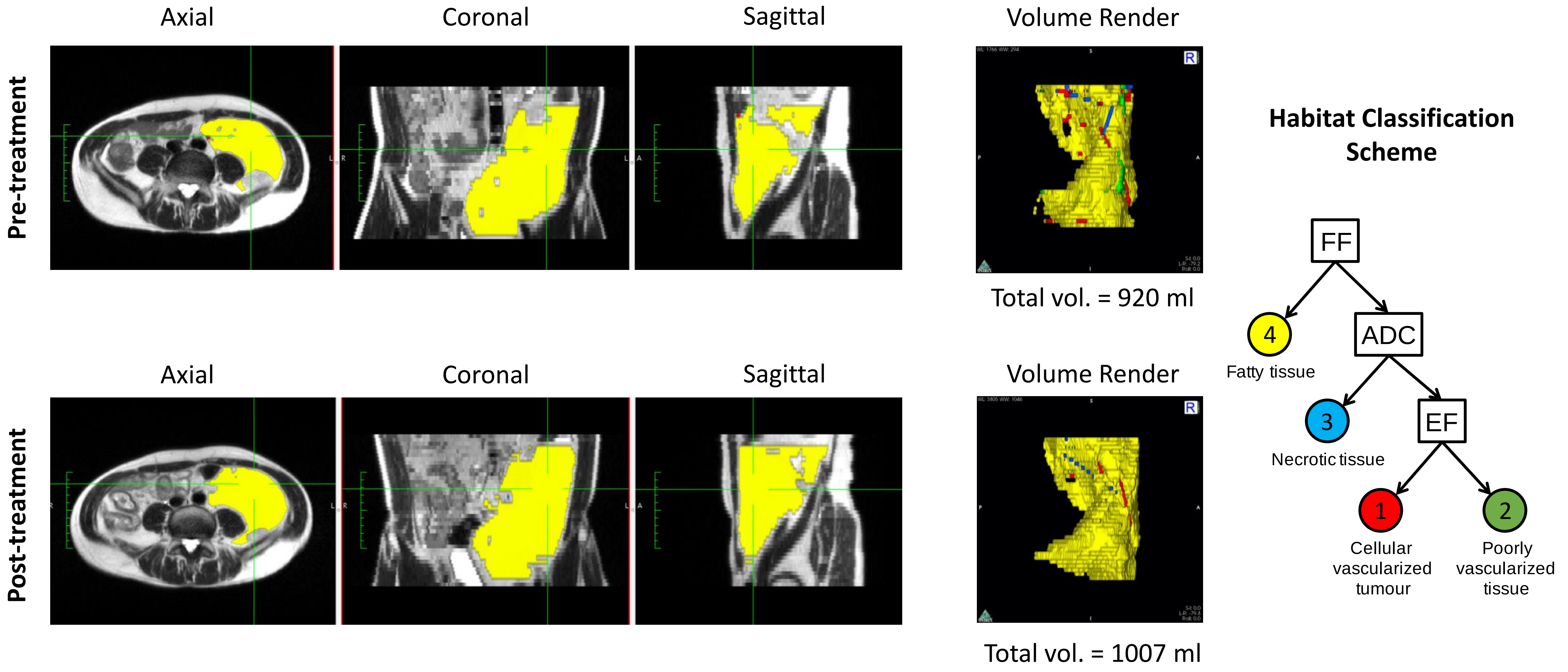
Axial
Coronal
Sagittal
Volume Render
Pre-treatment
Total vol. = 920 ml
Habitat Classification Scheme
FF
4
Fatty tissue
ADC
3
Necrotic tissue
EF
1
Cellular vascularized tumour
2
Poorly vascularized tissue
Axial
Coronal
Sagittal
Volume Render
Post-treatment
Total vol. = 1007 ml
