# Supplementary material for: Supervised Machine-Learning Enables Segmentation and Evaluation of Heterogeneous Post-treatment Changes in Multi-Parametric MRI of Soft-Tissue Sarcoma
Source: Front Oncol. 2019 Oct 10;9:941. doi: 10.3389/fonc.2019.00941 (PMC6795696; doi:10.3389/fonc.2019.00941)
Supplement: Supplementary file 4 [file Data_Sheet_4.PDF]

```python
"""
Author: Matthew D Blackledge

Copyright (c) 2017, The Institute of Cancer Research and The Royal Marsden.
All rights reserved.

Redistribution and use in source and binary forms, with or without
modification, are permitted provided that the following conditions are met:

* Redistributions of source code must retain the above copyright notice, this
list of conditions and the following disclaimer.

* Redistributions in binary form must reproduce the above copyright notice,
this list of conditions and the following disclaimer in the documentation
and/or other materials provided with the distribution.

* Neither the name of the copyright holder nor the names of its contributors
may be used to endorse or promote products derived from this software without
specific prior written permission.

THIS SOFTWARE IS PROVIDED BY THE COPYRIGHT HOLDERS AND CONTRIBUTORS "AS IS"
AND ANY EXPRESS OR IMPLIED WARRANTIES, INCLUDING, BUT NOT LIMITED TO, THE
IMPLIED WARRANTIES OF MERCHANTABILITY AND FITNESS FOR A PARTICULAR PURPOSE ARE
DISCLAIMED. IN NO EVENT SHALL THE COPYRIGHT HOLDER OR CONTRIBUTORS BE LIABLE
FOR ANY DIRECT, INDIRECT, INCIDENTAL, SPECIAL, EXEMPLARY, OR CONSEQUENTIAL
DAMAGES (INCLUDING, BUT NOT LIMITED TO, PROCUREMENT OF SUBSTITUTE GOODS OR
SERVICES; LOSS OF USE, DATA, OR PROFITS; OR BUSINESS INTERRUPTION) HOWEVER
CAUSED AND ON ANY THEORY OF LIABILITY, WHETHER IN CONTRACT, STRICT LIABILITY,
OR TORT (INCLUDING NEGLIGENCE OR OTHERWISE) ARISING IN ANY WAY OUT OF THE USE
OF THIS SOFTWARE, EVEN IF ADVISED OF THE POSSIBILITY OF SUCH DAMAGE.
"""

# Note: Please ensure that the extension is converted from '.txt' -> '.py' before
  use.

import numpy as np
from scipy.stats import multivariate_normal
from sklearn.neighbors import KernelDensity
from numpy import array as narray
import itertools
from collections import OrderedDict

class KDEClassifier(object):
    """
    Kernel Density Estimation classification.

    Performs supervised classfication of vector data into k chosen classes.

    Parameters
    ==========

    bandwidths : dictionary, optional (default=False)
                 A dictionary of bandwidths to apply for each expected class.
                 The keys should represent the classes for the given class
```

```
                 If a float is given, the same bandwidth will be used for each
class.
                 If None is given (default), then bandwdiths will computed using
the Silverman approximation.
                 Important: The classifier will normalise data to mean of 1 and
standard deviation 1.  The bandwidth
                            is applied after this transformation so may provide
unexpected results.

    background_class : object, optional (default=None)
                       The class that is expected to represent background noise.
                       All data with this class will be ignored in fitting
                       If set to None, then it is assumed there are no background
data

    Attrbiutes
    ==========

    background_class_ : object
                        The background class of the classifier (None if no
background fit)

    classes_ : array_like, shape (n_classes,)
              The unique classes used for this classifier

    bandwidths_ : dictionary
                  The n-vector of bandwidths used by the classifier (either set by
the user or calcaulted).

    kdes_ : dictionary
            The KernelDensity instances used for each class during fitting

    """

    def __init__(self, bandwidths=None, background_class=None):
        self.bandwidths_ = bandwidths
        self.background_class_=background_class
        self.classes_ = np.array([])
        self.kdes_ = OrderedDict()
        self.means_ = OrderedDict()
        self.stds_ = OrderedDict()
        self.volume_ = 1.0


    def fit(self, X, y):
        """
        Fit the KDE model according to the given training data

        Parameters
        ==========
        X : array-like, shape (n_samples, n_features)
            Training vectors, where n_samples is the number of samples and
            n_features is the number of features.
```

```
        y : array-like, shape (n_samples,)
            Target classes

        Returns
        =======
        self : object

        """

        # Get the unique classes
        self.classes_ = np.unique(y)
        if not self.background_class_ is None:
            if not self.background_class_ in self.classes_:
                self.classes_ = np.r_[self.classes_, self.background_class_]

        # For each class, determine the data mean and standard deviation
        self.means_ = OrderedDict()
        self.stds_ = OrderedDict()
        for l in self.classes_:
            if l == self.background_class_:
                self.means_[l] = None
                self.stds_[l]  = None
            else:
                data_l = X[y == l, :]
                self.means_[l] = np.mean(data_l, axis = 0)
                self.stds_[l] = np.std(data_l, axis=0) + 1e-16

        # For each class determine the bandwidth
        if self.bandwidths_ is None:
            self.bandwidths_ = OrderedDict()
            for l in self.classes_:
                if l == self.background_class_:
                    self.bandwidths_[l] = -1.0
                else:
                    self.bandwidths_[l] = np.power(4.0 / (3.0 * np.sum(1 * (y ==
l))), 0.2)

        # Train the KDEs for the model
        self.kdes_ = OrderedDict()
        for l in self.classes_:
            if l == self.background_class_:
                self.kdes_[l] = None
            else:
                kde = KernelDensity(bandwidth=self.bandwidths_[l])
                try:
                    kde.fit((X[y == l, :] - self.means_[l]) / self.stds_[l])
                except:
                    print self.bandwidths_
                    print l
                    print self.stds_
                    print X[y == l, :]
                    raise
                self.kdes_[l] = kde
```

```
        # Identify the range of the data
        self.volume_ = np.prod(np.max(X, axis = 0) - np.min(X, axis = 0))

        return self


    def predict_proba(self, X):
        """
        Compute probabilities of possible outcomes for samples in X.

        Parameters
        ==========
        X : array-like, shape (n_samples, n_features)
            The input data for which to calculate the probability

        Returns
        =======
        P : array-like, shape (n_samples, n_classes) or (n_samples, n_classes+1)
if background_class_ is True
            For each class, provides the The probability of each sample belonging
to each class
            The order of the probability can be gathered from the self.classes_
parameter

        """

        P = np.zeros((X.shape[0], len(self.classes_)))

        # Compute the absolute pdf values
        for i in range(len(self.classes_)):
            l = self.classes_[i]
            if l == self.background_class_:
                P[:, i] = 1.0 / self.volume_
            else:
                P[:, i] = np.exp(self.kdes_[l].score_samples((X - self.means_[l])
/ self.stds_[l])) / np.prod(self.stds_[l])

        # Return the a-priori probabilities
        return (P.T / np.sum(P, axis=1)).T

    def predict_log_proba(self, X):
        """
        Compute log-probabilities of possible outcomes for samples in X.

        Parameters
        ==========
        X : array-like, shape (n_samples, n_features)
           The input data for which to calculate the probability

        Returns
        =======
        P : array-like, shape (n_samples, n_classes) or (n_samples, n_classes+1)
if background_class_ is True
           For each class, provides the log-probability of each sample belonging
```

```
to each class
            The order of the probability can be gathered from the self.classes_
parameter

        """
        return np.log(self.predict_proba(X))

    def predict(self, X):
        """
        Predict the most likely class for each sample in X.

        Parameters
        ==========
        X : array-like, shape (n_samples, n_features)
            The input data for which to predict the classes

        Returns
        =======
        y_pred : array-like, shape (n_samples,)
                 Predicted class for each sample in X

        """

        P = self.predict_proba(X)
        arg_max = np.argmax(P, axis=1)
        labs = []
        for i in range(len(arg_max)):
            labs.append(self.classes_[arg_max[i]])
        return np.array(labs)

    def score(self, X, y):
        """
        Return the averaage classification accuracy of the classifier for the
given input data pair

        Parameters
        ==========
        X : array-like, shape (n_samples, n_features)
            Training vectors, where n_samples is the number of samples and
            n_features is the number of features.

        y : array-like, shape (n_samples,)
            Target classes

        Returns
        =======
        float : The score for the input pair

        """
        y_pred = self.predict(X)
        return float(np.sum(y_pred == y)) / len(y_pred)


class NBClassifier(object):
```

```
    """
    A naive Bayes classifier

    Performs supervised classfication of vector data into k chosen classes.

    Parameters
    ==========

    background_class : object, optional (default=None)
                       The class that is expected to represent background noise.
                       All data with this class will be ignored in fitting
                       If set to None, then it is assumed there are no background
data

    Attrbiutes
    ==========

    background_class : object
                        The background class of the classifier (None if no
background fit)

    classes_ : array-type
               The unique classes used for this classifier

    means_ : dictionary
              The mean values for each of the classes

    covariances_ : dictionary
                    The covariances for each of the classes

    weights_ : dictionary
               The weights for each of the classes
    """

    def __init__(self, background_class=None):
        self.background_class_ = background_class
        self.means_ = OrderedDict()
        self.covariances_ = OrderedDict()
        self.weights_ = OrderedDict()
        self.volume_ = 1.0
        self.classes_ = np.array([])

    def fit(self, X, y):
        """
        Fit the naive Bayes model according to the given training data

        Parameters
        ==========
        X : array-like, shape (n_samples, n_features)
            Training vectors, where n_samples is the number of samples and
            n_features is the number of features.

        y : array-like, shape (n_samples,)
            Target classes
```

```
        Returns
        =======
        self : object

        """

        self.classes_ = np.unique(y)
        if not self.background_class_ is None:
            if not self.background_class_ in self.classes_:
                self.classes_ = np.r_[self.classes_, self.background_class_]

        self.means_ = OrderedDict()
        self.covariances_ = OrderedDict()
        self.weights_ = OrderedDict()

        for l in self.classes_:
            if l == self.background_class_:
                self.means_[l] = None
                self.covariances_[l] = None
                self.weights_[l] = None
            else:
                data_l = X[y == l, :]
                self.means_[l] = np.mean(data_l, axis = 0)
                if len(data_l) == 1:
                    self.covariances_[l] = 1e30
                else:
                    self.covariances_[l] = np.cov(data_l.T)
                self.weights_[l] = float(np.sum(1 * (y == l)))/len(y)

        # Identify the range of the data
        self.volume_ = np.prod(np.max(X, axis=0) - np.min(X, axis=0))

        return self


    def predict_proba(self, X):
        """
        Compute probabilities of possible outcomes for samples in X.

        Parameters
        ==========
        X : array-like, shape (n_samples, n_features)
           The input data for which to calculate the probability

        Returns
        =======
        P : array-like, shape (n_samples, n_classes) or (n_samples, n_classes+1)
if background_class_ is True
            For each class, provides the The probability of each sample belonging
to each class
            The order of the probability can be gathered from the self.classes_
parameter
```

```
        """

        # Compute the absolute pdf values

        P = np.zeros((X.shape[0], len(self.classes_)))

        for i in range(len(self.classes_)):
            l = self.classes_[i]
            if l == self.background_class_:
                P[:, i] = 1.0/self.volume_
            else:
                P[:, i] = multivariate_normal.pdf(X, mean = self.means_[l], cov = 
self.covariances_[l],
                                                          allow_singular=True)

        # Return the a-priori probabilities
        return (P.T / np.sum(P, axis=1)).T

    def predict_log_proba(self, X):
        """
        Compute log-probabilities of possible outcomes for samples in X.

        Parameters
        ==========
        X : array-like, shape (n_samples, n_features)
            The input data for which to calculate the probability

        Returns
        =======
        P : array-like, shape (n_samples, n_classes) or (n_samples, n_classes+1) 
if background_class_ is True
            For each class, provides the log-probability of each sample belonging 
to each class
            The order of the probability can be gathered from the self.classes_ 
parameter

        """
        return np.log(self.predict_proba(X))

    def predict(self, X):
        """
        Predict the most likely class for each sample in X.

        Parameters
        ==========
        X : array-like, shape (n_samples, n_features)
            The input data for which to predict the classes

        Returns
        =======
        y_pred : array-like, shape (n_samples,)
                 Predicted class for each sample in X

        """
```

```
        P = self.predict_proba(X)
        max_idx = np.argmax(P, axis=1)
        labs = []
        for i in range(len(max_idx)):
            labs.append(self.classes_[max_idx[i]])
        return np.array(labs)

    def score(self, X, y):
        """
        Return the averaage classification accuracy of the classifier for the
given input data pair

        Parameters
        ==========
        X : array-like, shape (n_samples, n_features)
            Training vectors, where n_samples is the number of samples and
            n_features is the number of features.

        y : array-like, shape (n_samples,)
            Target classes

        Returns
        =======
        float : The score for the input pair

        """
        y_pred = self.predict(X)
        return float(np.sum(y_pred == y)) / len(y_pred)


class MRFClassifier(object):
    """
    Markov Random Field classification.

    Performs iterated conditional modes smoothing of classification results from
    a supervised classifier optimised over a D-dimensional image.

    Parameters
    ==========

    clf : object
          A valid ML classifier such as the ones given in this module or those
provided by the Scikit-Learn package
          The classifier must be able to respond to the signature
clf.predict_log_proba(X) at a minumum
          This clf instance must have already been fit!

    beta : float, optional (default=1)
           The neighborhood prior for the MRF.  Set to 0 for no neighborhood prior
or higher for more
           bias towards neighbors being similar

    iter_max : int, optional (default = 10)
               The maximum number of ICM iterations to perform
```

```
    neighborhood : array-like, shape (n_neighbors, D), optional (default=None)
                   The neighborhood over which to compute the MRF.
                   Default is neighborhood = [[-1,0], [0,-1], [1,0], [0,1],
[-1,1], [1,-1], [1,1], [-1,-1]]

    Attrbiutes
    ==========

    cur_iter : int
              The current iteration of the ICM algorithm

    """
    def __init__(self, clf, beta = 1.0, iter_max = 10, neighbourhood = None, eps =
0):
        self.clf = clf
        self.beta = beta
        self.iter_max = iter_max
        self.iter = 0
        self.eps = eps
        self.cost = 1e40
        self.neighborhood = neighbourhood
        self._shape = ()

    def predict_icm(self, X, mask = None, callback = None):
        """
        Predict the class classes for an input image using an Iterated Conditional
Modes (ICM) algorithm.

        Parameters
        ==========
        X : array-like, shape (n1, n2, ..., nD, n_features):
            The image data over which to predict the final classification
            n1, n2, ..., nD represent the spatial dimensions of the image
            n_features is the number of features per image voxel (must match
number of features expected by the classifier)

        mask : array-like (dtype=bool), shape (n1, n2, ..., nD), optional
(default=None)
              The mask over which to calculate the classes.
              If equal to None, then ICM is computed over all voxels.

        callback : ufunc, optional (default=None)
                   If provided, the input function will be called after each ICM
loop
                   The function must expect a single input, which will be the
instance of this class.
                   If equal to None, then no callback function is used.

        Returns
        =======
        y_pred: array-like, shape (n1, n2, ..., nD)
               Predicted class for each sample in X
```

```
        """

        if self.neighborhood is None:
            self.neighborhood = np.array([[-1,0], [0,-1], [1,0], [0,1], [-1,1],
[1,-1], [1,1], [-1,-1]])

        dims = len(X.shape) - 1
        imshape = np.array(X.shape[0:-1])
        if mask is None:
            mask = np.ones(imshape, dtype='bool')
        else:
            if not np.array_equal(mask.shape, X.shape[0:-1]):
                raise Exception(ValueError, "Input shape of data and mask do not
match")

        data = X.reshape([np.prod(imshape), X.shape[-1]])
        mask_idx = mask.ravel()

        # Create an array of log likelihoods over the image
        log_likelihood = self.clf.predict_log_proba(data)
        n_unique_classes = len(self.clf.classes_)
        log_likelihood = log_likelihood.reshape(np.r_[imshape, n_unique_classes])

        # Create an array of classes over the image
        # Where no mask is present, a class of None is given.
        l = self.clf.predict(data[mask_idx])
        classes = np.zeros(imshape, dtype = 'object')
        classes[:] = None
        classes[mask] = l

        iterator = []
        for i in range(dims):
            iterator.append(range(imshape[i]))
        iterator = itertools.product(*iterator)
        iterator = list(iterator)

        if not callback is None:
          callback(self)

        for self.iter in range(1, self.iter_max+1):
            classes_old = classes.copy()
            for idx in iterator:
                if mask[tuple(idx)] == True:
                    neighbors = [classes[tuple(i + idx)]
                                 if mask[tuple((i + idx) % imshape)]
                                 and np.min(i+idx) >= 0
                                 and np.sum(i+idx >= imshape) == 0
                                 else None for i in self.neighborhood]
                    lp = []
                    for l in self.clf.classes_:
                        lp.append(self.beta * np.sum(neighbors == l))
                    lp = np.array(lp) + log_likelihood[idx]
                    classes[tuple(idx)] = self.clf.classes_[np.argmax(lp)]
```

```
            # If the algorithm has converged then exit
            self.cost = np.sum(np.abs(classes[mask] - classes_old[mask]) > 0)
            if self.cost <= self.eps:
                break

            self.clf.fit(data[mask_idx, :], classes.ravel()[mask_idx])
            log_likelihood = self.clf.predict_log_proba(data)
            n_unique_classes = len(self.clf.classes_)
            log_likelihood = log_likelihood.reshape(np.r_[imshape,
n_unique_classes])

            if not callback is None:
                callback(self)

        return classes

class ROIResult(object):
    """
    A container for a single supervised ROI instance within the dataset

    Parameters
    ==========

    patid : str
            The patient ID

    roitype : int
             The class of the ROI (1, 2, 3 or 4)

    ADC : array-like
          The ADC values associated with the ROI

    EF : array-like
         The enhancement fraction values associated with the ROI

    FF : array-like
         The fat-fraction values associate with the ROI

    """

    def __init__(self, ID, roitype, ADC, EF, FF):
        self.ID = ID
        self.roitype = roitype
        self.ADC = ADC
        self.EF = EF
        self.FF = FF

    def __str__(self):
        return "ROI with name %s and type %s"%(self.ID, self.roitype)

    def __repr__(self):
        return "<ROIResult, %s, %d>"%(self.ID, self.roitype)
```

```python
class ROIResultList(list):
    """
    A container for a list of ROIResult instances.
    Performs all required data stratification

    """

    def __str__(self):
        str = "ROIResultList: %d ROI objects as follows:\n"%len(self)
        for roi in self:
            str+="%s, %d\n"%(roi.ID, roi.roitype)
        return str

    def shuffle(self):
        """
        Shuffle the contained data

        Returns
        =======
        self : object

        """
        idx = np.arange(len(self))
        np.random.shuffle(idx)
        temp = []
        for i in range(len(self)):
            temp.append(self[idx[i]])
        for i in range(len(self)):
            self.__setitem__(i, temp[i])

        return self

    def listwithid(self, ID):
        """
        Return a list of ROIResult instances with a given ID

        Parameters
        ==========

        ID : str
             The patient ID

        Returns
        =======
        list : The list of ROIResult instances with the given ID

        """
        temp = []
        for i in range(len(self)):
            if self[i].ID == ID:
                temp.append(self[i])
        return temp

    def listwithtype(self, roitype):
```

```python
        """
        Return a list of ROIResult instances with a given ROI type (0, 1, 2, 3, 4)

        Parameters
        ==========

        roitype : int
                  The ROI type

        Returns
        =======
        list : The list of ROIResult instances with the given ROI type

        """
        temp = []
        for i in range(len(self)):
            if self[i].roitype == roitype:
                temp.append(self[i])
        return temp

    def arraywithtype(self, roitype):
        """
        Return a list of ROIResult instances with a given ROI type (0, 1, 2, 3, 4)

        Parameters
        ==========

        roitype : int
                  The ROI type

        Returns
        =======
        list : The list of ROIResult instances with the given ROI type

        """

        return np.array(self.listwithtype(roitype))

    def removerois(self, rois):
        """
        Return a ROIResultList with the rois in 'rois' removed

        Returns
        =======
        ROIResultList : The smaller roi list

        """
        rois_smaller = []
        for roi in self:
            if not roi in rois:
                rois_smaller.append(roi)
        return ROIResultList(rois_smaller)

    def types(self):
```

```
        """
        Return a list of contained ROIResult types

        Returns
        =======
        list : The list of ROIResult types

        """
        types = []
        for r in self:
            types.append(r.roitype)
        return narray(types)

    def split(self, p):
        """
        Split the list of ROIResult instances by some fraction
        This process is not random, the returned values will contain the values:
        self[0:p*len(self)] and self[p*len(self)::]

        Parameters
        ==========

        p : float
            The fraction by which to split the results

        Returns
        =======
        (ROIResultList, ROIResultList) : The Split results

        """
        if p <= 0 or p >= 1:
            raise Exception(ValueError, "Split proportion should be 0 <= p <= 1")

        s = len(self)
        n = int(np.floor(p*s))
        return ROIResultList(self[0:n]), ROIResultList(self[n::])

    def stratified_loo(self, ignore_types = None):
        """
        Perform a stratified leave-one-out (loo).
        This will split the contained ROIResult instances into two groups:
        1. One ROIResult from each type (unless the type is ignored via the
           'ignore_types' parameter.
        2. The remaining results after those from (1) have been removed.

        Parameters
        ==========

        ignore_types : boolean, optional (default=None)
                       The types that should be ignored in the loo

        Returns
        =======
        (ROIResultList, ROIResultList) : The Split results, the first containing
```

```
the loo
                                      results, the second the remaining data

        """
        remain = []
        for i in range(len(self)):
            remain.append(self[i])
        t = self.types()
        loos = []
        idx = np.arange(len(self))
        for i in np.unique(t):
            if ignore_types:
                if i in ignore_types:
                    continue
            idx_ = np.random.choice(idx[t == i])
            r = self[idx_]
            loos.append(r)
            remain.remove(r)
        return ROIResultList(loos), ROIResultList(remain)

    def getobservations(self):
        """
        Get a nd-array containing the contained quantitative data

        Returns
        =======
        array-like : shape (n_samples, 3)
                     The contained ROI values in order of ADC, FF, then EF

        array-like : shape (n_samples,)
                     The associated ROI types for the data

        """
        ADCs = narray([])
        FFs = narray([])
        EFs = narray([])
        types = narray([])
        for r in self:
            ADCs = np.r_[ADCs, r.ADC]
            FFs = np.r_[FFs, r.FF]
            EFs = np.r_[EFs, r.EF]
            types = np.r_[types, np.repeat(r.roitype, len(r.ADC))]
        return np.c_[ADCs, EFs, FFs], types

    def getrescaledobservations(self):
        """
        Get a nd-array containing the contained quantitative data with each value
        normalised to the range [0,1] (ignoring noise effects -> some values maybe
outside this range)

        Returns
        =======
        array-like : shape (n_samples, 3)
                     The contained ROI values in order of ADC, FF, then EF
```

```
        array-like : shape (n_samples,)
                    The associated ROI types for the data

        """
        vals, types = self.getobservations()
        vals[:, 0] = vals[:, 0]/3000
        vals[:, 1] = (vals[:, 1] + 1000.0) / 2000
        vals[:, 2] = vals[:, 2]/1000
        return vals, types

def score_estimator_single_class(true_labels, estimated_labels):
    """
    Score an estimator for label assignment.
    Scores the estimator for each label it tries to classify

    Parameters
    ==========
    true_labels : ndarray
        The true labels that the estimator should classify
    estimated_labels : ndarray
        The estimated labels

    Returns
    =======
    result : dict
    A dictionary containing the percentage of time the estimator has accurately
predicted the label
    (each label seperately)
    """

    true_labels = np.array(true_labels)
    estimated_labels = np.array(estimated_labels)

    unique_labels = np.unique(true_labels)

    result = {}
    for l in unique_labels:
        idx = true_labels == l
        n_labels = np.sum(idx)
        n_correct = np.sum(1.0 * (true_labels[idx] == estimated_labels[idx]))
        result[l] = float(n_correct) / n_labels * 100.0

    return result

def score_estimator_single_class_as_list(true_labels, estimated_labels):
    """
    Score an estimator for label assignment.
    Scores the estimator for each label it tries to classify

    Parameters
    ==========
    true_labels : ndarray
        The true labels that the estimator should classify
```

```
    estimated_labels : ndarray
        The estimated labels

    Returns
    =======
    result : list
    A list containing the percentage of time the estimator has accurately
predicted the label
    (each label seperately)
    """

    result = score_estimator_single_class(true_labels, estimated_labels)

    return [result[i] for i in [0, 1, 2, 3, 4]]

def score_estimator_all(true_labels, estimated_labels):
    """
    Score an estimator for label assignment.
    Scores the estimator for all labels it tries to classify

    Parameters
    ==========
    true_labels : ndarray
        The true labels that the estimator should classify
    estimated_labels : ndarray
        The estimated labels

    Returns
    =======
    result : float
    The percentage of time the estimator has accurately predicted the labels
    """
    return float(np.sum(estimated_labels == true_labels)) / len(true_labels) *
100.0
```
